# Supplementary figures and images for: Disease modeling and pharmacological rescue of autosomal dominant retinitis pigmentosa associated with RHO copy number variation
Source: eLife. 2024 Apr 25;12:RP90575. doi: 10.7554/eLife.90575 (PMC11045220; doi:10.7554/eLife.90575)

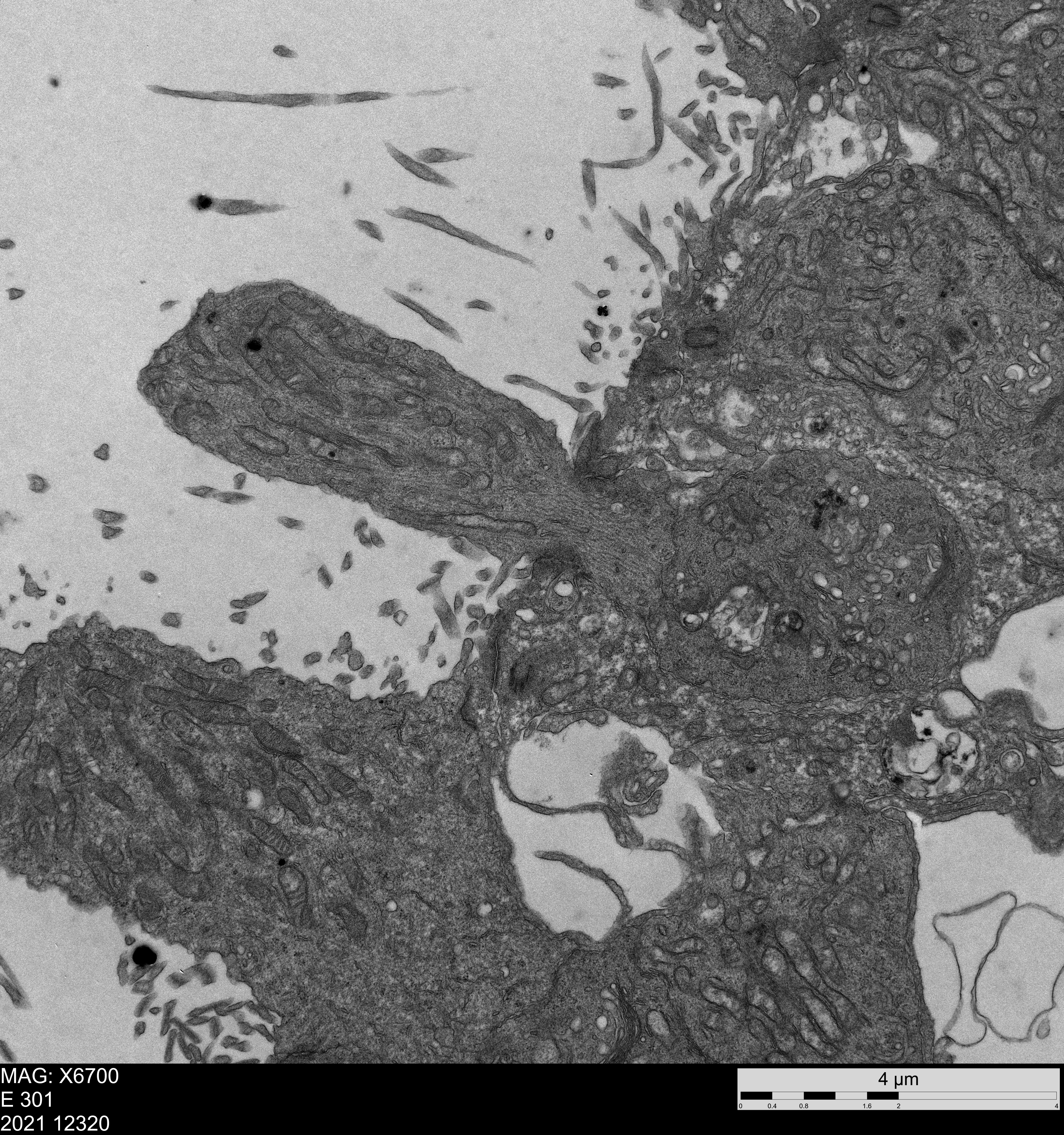

Supplement: Figure 2—source data 1. [file elife-90575-fig2-data1.zip › Fig2/E 301-030122 Lamba-SK-12320.tif]

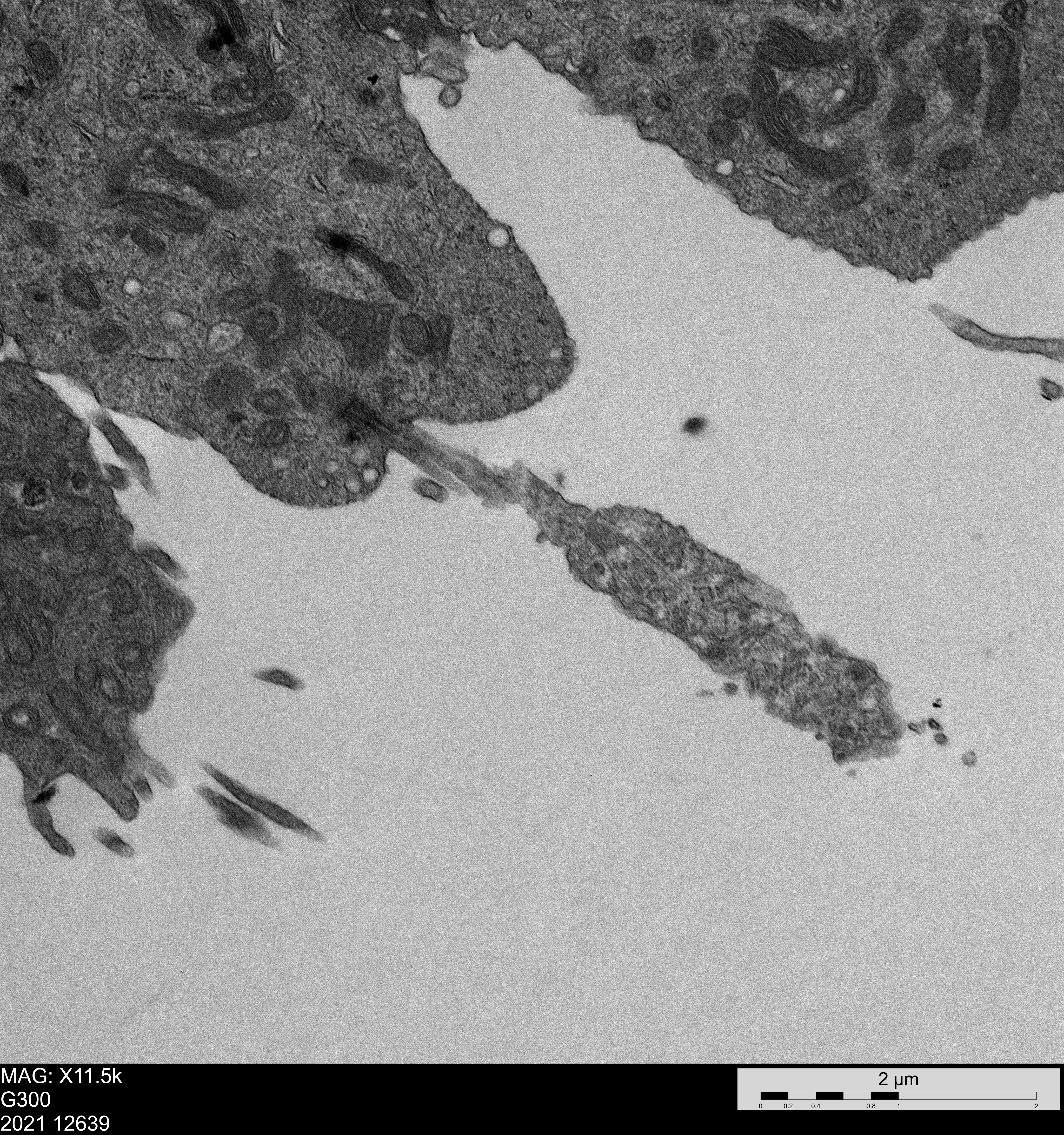

Supplement: Figure 2—source data 1. [file elife-90575-fig2-data1.zip › Fig2/G300-030122Lamba-SK-12639.tif]

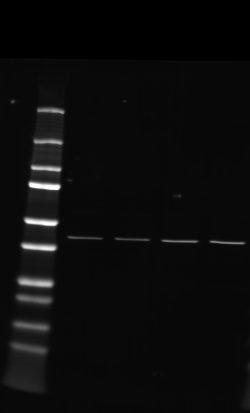

Supplement: Figure 4—source data 1. [file elife-90575-fig4-data1.zip › Fig4/WesternBlot/Actin_for_RHO_RotatedCW180_22-45_1-19-23_700.tif]

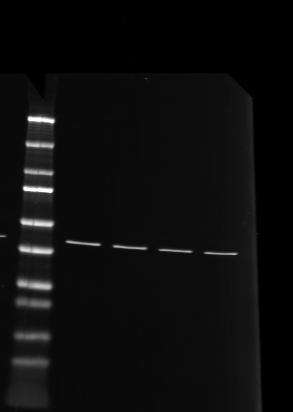

Supplement: Figure 4—source data 1. [file elife-90575-fig4-data1.zip › Fig4/WesternBlot/Actin_for_SAG_RotatedCW180_22-45_1-19-23_700.tif]

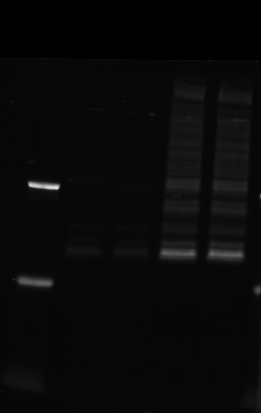

Supplement: Figure 4—source data 1. [file elife-90575-fig4-data1.zip › Fig4/WesternBlot/RHO-RotatedCW180_22-45_1-19-23_800.tif]

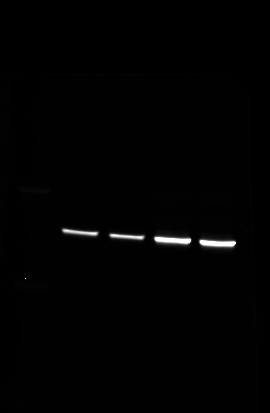

Supplement: Figure 4—source data 1. [file elife-90575-fig4-data1.zip › Fig4/WesternBlot/SAG-RotatedCW180_22-45_1-19-23_800.tif]
